# The Use of Microbial Accessible and Fermentable Carbohydrates and/or Butyrate as Supportive Treatment for Patients With Coronavirus SARS-CoV-2 Infection

**DOI:** 10.3389/fmed.2020.00292

**Published:** 2020-06-05

**Authors:** Douglas L. Archer, Dean C. Kramer

**Affiliations:** ^1^Food Science and Human Nutrition Department, Institute of Food and Agricultural Science, University of Florida, Gainesville, FL, United States; ^2^Gastroenterology, Kramer Medical Clinic, Gainesville, FL, United States

**Keywords:** SCFAs, butyrate, adjunct therapy, cytokine storm, anti-inflammatory

In addition to symptoms of fever, cough, and dyspnea, SARS-CoV-2 infection has been reported to also produce gastrointestinal symptoms. These symptoms can include anorexia, nausea, vomiting, and diarrhea. They are not unexpected symptoms, since enterocytes express the ACE2 receptor, and other requisite enzymes for SARS-CoV-2 uptake and replication (1). Viral infection leading to enterocyte death would predictably cause both malabsorption and increased G.I. permeability (2). Up to 50% of coronavirus cases reported from China had G.I. related symptoms (3), with recent corroboration in the United States (4).

After exposure to SARS-CoV-2, the estimated lag before onset of symptoms averages about 5 days. With limited oral intake following the onset of symptoms due to anorexia and other GI perturbations, there is marked alteration in intestinal microbial composition (dysbiosis) resulting in both macro and micronutrient deficiencies (5). The alterations include a decrease in carbohydrate fermentation and precipitous decline in production of short chain fatty acids, most notably butyrate.

Butyrate represents a well-characterized short chain fatty acid and functions as a key modulator of the immune system both in the intestinal tract (6) as well as in the lungs (7), and is instrumental in maintaining the physical integrity and balanced permeability of the gut mucosal lining (8).

Deficiency of short chain fatty acids negatively impacts energy balance resulting in failure of the intestinal epithelial barrier and associated immune defenses. With increased intestinal damage and permeability, pathogenic microbes, their components, and other foreign antigens may enter the body unimpeded. It is, therefore, not surprising that damage to distant organs appears in the heart, liver, kidney, and brain as epithelial barriers collapse and inflammation processes intensify.

Additionally, with lack of nutrients, short chain fatty acids are no longer available to stimulate bone marrow hematopoeisis which is felt to be a critical part of the gut-bone marrow-lung axis. Airway inflammation (7), the hallmark of acute respiratory distress syndrome (ARDS), proceeds unchecked frequently leading to death.

The short chain fatty acid butyrate benefits patients with established allergic lung inflammation (9). Specifically, butyrate has been shown to affect eosinophil trafficking and is able to “blunt migration into the lung, to reduce airway eosinophilia, and to ameliorate impaired lung function” (9).

Under siege by SARS-CoV-2, and perhaps due to over-stimulation of the gut associated lymphoid tissue, host defenses launch a counterattack releasing massive amounts of cytokines, resulting in a “*cytokine storm”* (10). The cytokine storm attempts to destroy the infecting virus, but, in the process, collateral damage to nearby tissue occurs as well. With SARS-CoV-2 this is particularly true in lung tissue (11). Given that the G.I. tract is the body's largest immunologic organ, it may be, in part, the genesis of the cytokine storm.

The inflammation accompanying SARS-CoV-2 infection is reflected by high blood levels of C-reactive protein, TNF-alpha, and several interleukins, most notably proinflammatory IL-6 (12). Elevated IL-6 in severe Covid-19 patients is a predictor of higher mortality rates (13). Some successes have been reported after treatment of Covid-19 patients with anti-inflammatory agents such as prednisone, and other anti-inflammatory drugs, including the IL-6 antagonist Tocilizumab (12). Butyrate has definitively been found to lower IL-6 levels in both animal and human studies (14, 15).

Conceptually, providing the necessary substrate for the continued production of short chain fatty acids using supplements offers an appealing approach to minimizing the damage caused by SARS-CoV-2. Fructooligosaccharides, arabinoxylose, galactooligosaccharides, and gum guar represent but a few of the readily available carbohydrates capable of fermentation and production of short chain fatty acids. Gastric infusion of short chain fatty acids has been shown in the experimental animal to improve intestinal barrier function (16). The effect of short chain fatty acids, especially butyrate, on gastrointestinal function in animals has been extensively studied with mixed findings, although recent modifications of formulations that modify the release profile have shown superior results (17).

Supplementing SARS-CoV-2 infected patients who are capable of tolerating oral intake with microbial accessible and fermentable carbohydrates could serve as a helpful adjunct in treating SARS-CoV-2 infection. For those patients unable to take food supplements or liquids by mouth, or who display symptoms of gastric upset or dysbiosis, nasogastric gavage of a butyrate solution or rectal administration of butyrate by enema could be considered. The use of butyrate enemas has been reported for multiple conditions including ulcerative colitis, diversion colitis, radiation proctitis, and pouchitis. Studies have yielded varying results making interpretation difficult [reviewed in Hamer et al. (18), Luceri et al. (19)]. The studies reported suffer from the lack of a standard protocol such as varying enema volumes, concentrations of butyrate, and frequency of administration.

The proposed use of butyrate by enema would accomplish two things:

- Direct application of butyrate to the site of the intestinal tract, terminal ileum and right colon that contains one of the highest concentrations of SARS-CoV-2 receptors, and
- Increase butyrate absorption for systemic distribution since the colon is the primary site for both production and absorption of butyrate.

Butyrate therapy has been demonstrated safe when administered by enema (18), per os (20), or intravenously (21). More research into these potential adjunct therapies should be encouraged.

## Author Contributions

The authors DK and DA contributed equally to this work and approved it for publication.

## Conflict of Interest

The authors declare that the research was conducted in the absence of any commercial or financial relationships that could be construed as a potential conflict of interest.
